# Psychometric properties of the Centers for Disease Control and Prevention Health-Related Quality of Life (CDC HRQOL) items in adults with arthritis

**DOI:** 10.1186/1477-7525-4-66

**Published:** 2006-09-24

**Authors:** Thelma Mielenz, Elizabeth Jackson, Shannon Currey, Robert DeVellis, Leigh F Callahan

**Affiliations:** 1 Thurston Arthritis Research Center, School of Medicine, University of North Carolina at Chapel Hill, USA; 2 Division of Physical Therapy, School of Medicine, University of North Carolina at Chapel Hill, USA; 3 Department of Health Behavior & Health Education, School of Public Health, University of North Carolina at Chapel Hill, USA; 4 Departments of Medicine, Orthopaedics and Social Medicine, School of Medicine, University of North Carolina at Chapel Hill, USA

## Abstract

**Background:**

Measuring health-related quality of life (HRQOL) is important in arthritis and the SF-36v2 is the current state-of-the-art. It is only emerging how well the Centers for Disease Control and Prevention (CDC) HRQOL measures HRQOL for people with arthritis. This study's purpose is to assess the psychometric properties of the 9-item CDC HRQOL (4-item Healthy Days Core Module and 5-item Healthy Days Symptoms Module) in an arthritis sample using the SF-36v2 as a comparison.

**Methods:**

In Fall 2002, a cross-sectional study acquired survey data including the CDC HRQOL and SF-36v2 from 2 North Carolina populations of adult patients reporting osteoarthritis, rheumatoid arthritis, and fibromyalgia; 2182 (52%) responded. The first item of both the CDC HRQOL and the SF-36v2 was general health (GEN). All 8 other CDC HRQOL items ask for the number of days in the past 30 days that respondents experienced various aspects of HRQOL. Exploratory principal components analyses (PCA) were conducted on each sample and the combined samples of the CDC HRQOL. The multitrait-multimethod matrix (MTMM) was used to compute correlations between each trait (physical health and mental health) and between each method of measurement (CDC HRQOL and SF36v2). The relative contribution of the CDC HRQOL in predicting the physical component summary (PCS) and the mental component summary (MCS) was determined by regressing the CDC HRQOL items on the PCS and MCS scales.

**Results:**

All 9 CDC HRQOL items loaded primarily onto 1 factor (explaining 57% of the item variance) representing a reasonable solution for capturing overall HRQOL. After rotation a 2 factor interpretation for the 9 items was clear, with 4 items capturing physical health (physical, activity, pain, and energy days) and 3 items capturing mental health (mental, depression, and anxiety days). All of the loadings for these two factors were greater than 0.70. The CDC HRQOL physical health factor correlated with PCS (r = -.78, p < 0.0001) and the mental health factor correlated with MCS (r = -.71, p < 0.0001). The relative contribution of the CDC HRQOL in predicting PCS was 73% (R^2 ^= .73) when GEN was included in the CDC HRQOL score and 65% (R^2 ^= .65) when GEN was removed. The relative contribution of the CDC HRQOL in predicting MCS was 56% (R^2 ^= .56) when GEN was included and removed.

**Conclusion:**

The CDC HRQOL appears to have strong psychometric properties in individuals with arthritis in both community-based and subspecialty clinical settings. The 9 item CDC HRQOL is a reasonable measure for overall HRQOL and the two subscales, representing physical and mental health, are reasonable when the goal is to examine those aspects.

## Background

Measures of health status, including quality of life, have become increasingly important considerations in decisions regarding resource allocation, intervention design, and treatment of individuals with chronic disease. Researchers have conceptualized quality of life on many levels, and there are multiple views on how it should be defined and measured [1]. The health community has generally chosen to focus on the individual-level aspects of quality of life that can be shown to affect physical and mental health. This narrower concept is referred to as health-related quality of life (HRQOL).

The Medical Outcomes Study Short Form (SF-36) is a well-known, extensively researched, self-report HRQOL measure studied in a variety of populations by Ware and colleagues [2]. In a study published in 2004, the SF-36 was used to evaluate the impact of chronic conditions on HRQOL in eight countries [3]. Arthritis was found to have the greatest effect on HRQOL through the physical component summary (PCS) of the SF-36 [3]. Two studies evaluated adult patients with arthritis in four clinical trials with the overall objective of testing the psychometric properties of the SF-36 in this population. These studies conclude that the SF-36 is a psychometrically sound tool in adults with arthritis [4,5].

In 1993, the Centers for Disease Control and Prevention (CDC) developed a 4-item Healthy Days Core Module as a tool for public health surveillance of HRQOL [6]. These items are included in the Behavioral Risk Factor Surveillance System (BRFSS) currently being used in all states, the District of Columbia, and three territories, as well as in the National Health and Nutrition Examination Survey (NHANES). The CDC added the 5-item Healthy Days Symptoms Module and 5-item Activity Limitation Module as optional modules in 1995 [6].

The broad objective of this study is to assess the psychometric properties of the 9-item CDC HRQOL (4-item Health Days Core Module and 5-item Healthy Days Symptoms Module) in two samples of individuals with arthritis, a community-based sample and a subspecialty sample, using the SF-36v2 as a comparison. This objective was addressed by: 1) examining the underlying factor structure of the CDC HRQOL items cross-sectionally in the two samples; 2) assessing the extent and nature of shared variation in the identified CDC HRQOL items and the SF-36v2 physical health component summary (PCS) score and mental health component summary (MCS) score; and 3) determining the relative contribution of the components of the CDC HRQOL items in predicting the SF-36v2 scores.

## Methods

### Sample

In fall 2002, a self-report survey, including the 2 sets of HRQOL measures (the SF-36v2 and the 9-item CDC HRQOL) was mailed to 4183 patients with osteoarthritis (OA), rheumatoid arthritis (RA), and/or fibromyaligia (FM). Two populations of individuals with arthritis: the North Carolina Family Practice Resource Network (NC-FP-RN) and the musculoskeletal database (MSK) were combined for this study. This study was approved by the University of North Carolina (UNC) Institutional Review Board (IRB).

### North Carolina Family Practice Resource Network

The NC-FP-RN is a practice-based, university-sponsored network devoted to research on chronic diseases in primary care [7]. The NC-FP-RN enrolled 4760 individuals (62% of eligible patients) from 17 family practice sites dispersed across North Carolina in 2001. Enrollees completed a self-report questionnaire assessing health status and chronic conditions. Informed consent was obtained and participants confirmed their approval to be contacted for future studies. There were 2182 individuals who self-reported OA, RA, FM, or reported any symptoms of pain, aching, or stiffness in or around a joint in the past thirty days. These participants were selected for this study.

### Musculoskeletal database

The second population was the MSK database. Consecutive adult patients are enrolled from musculoskeletal subspecialty clinics, including: 1) rheumatology, orthopaedics, spine, and sports medicine clinics from the University of North Carolina teaching hospital, and 2) thirteen community rheumatology clinics in North Carolina. The MSK database was started in 1995. Participants who agree to participate in the MSK database complete a baseline self-report health status questionnaire and consent to be contacted for future studies. The participants' physicians provided diagnosis at the time of study enrollment. Periodically individuals in the database with selected diagnoses are mailed self-report questionnaires designed to assess health outcomes. Participants with the three most common rheumatic disease diagnoses (RA, OA, and FM) were selected, resulting in a subsample of 2001 adults for this study.

From the NC-FP-RN, 1139 participants completed surveys in 2002. After deceased participants (n = 27) and incorrect addresses (n = 194) were subtracted from the denominator (n = 2182), the response rate was 58%. In the MSK population, 681 participants completed surveys with a 43% response rate after subtracting the known deceased participants (n = 20) and incorrect addresses (n = 390). Given that it was established in 1995, it is possible that the MSK database contained patients whose deceased status was undetected. The response rate was 51% when combining the completed surveys from both populations. The non-respondents were significantly younger, less educated, and more likely to be black or other race than white when compared to the respondents. The overall response rate was only fair and may not be representative of all people with arthritis. However, in psychometric analyses response rates are less important than in other analytical studies, because instruments are validated on specific populations [8].

### Measures

#### SF-36v2

The Medical Outcomes Study Short Form version 2 SF-36v2 is a 36-item questionnaire that assesses general health status by eight health attributes, including: physical functioning, social functioning, role limitations due to physical health, role limitations due to emotional problems, mental health, vitality, bodily pain, and general health perceptions [9]. Each attribute is a multi-item scale containing 2–10 items each with Likert scales. These attributes are combined using a regression equation and standardized to population norms to provide a physical component summary (PCS) and a mental component summary (MCS). The SF-36v2 is scored 0–100 with higher scores indicating better health status [9].

#### CDC HRQOL

Nine items from the CDC's measure of HRQOL (the CDC HRQOL) were used. These items included the 4-item Healthy Days Core Module and the 5-item Healthy Days Symptoms Module. The CDC 4-item Healthy Days Core Module scores range from 0 to 30, with higher scores indicating worse perceived health. Item one assesses self-rated general health with five responses ranging from excellent to poor. This item is identical to the first question of the SF-36v2. All other eight CDC HRQOL items ask about the number of days in the past 30 days the patient has experienced particular health-related conditions or problems. Items two and three assess the number of days in the past 30 of impaired physical or mental health, and item four assesses recent limitations in usual activities due to poor physical or mental health. The summary index of unhealthy days is calculated by summing the responses to the physically unhealthy and mentally unhealthy days (items two and three). If the sum is greater than 30, a maximum score of 30 is assigned. This summary index of unhealthy days assumes a minimal logical overlap of reported physically and mentally unhealthy days [6,10].

The CDC 5-item Healthy Days Symptoms Module asks the number of recent days for the following 5 symptoms: 1) pain 2) depression 3) anxiety 4) sleeplessness and 5) energy. Other than the "unhealthy days" summary index described above, the items are typically not combined; all items were treated separately in these analyses. For eight items, including the general health item, higher scores mean worse problems. For the lack of energy item from the Healthy Days Symptoms Module, scored 0 to 30, higher scores indicate better health.

### Procedure

The order of the SF-36v2 and the CDC HRQOL items was alternated to decrease ordering effects. Participants were mailed the survey, then a postcard, and then a second mailing. Follow-up phone calls to non-respondents were done in the NC-FP-RN to increase the response rate because this sample was also being used for another study; follow-up phone calls were not done in the MSK database.

### Data analysis

The following analyses were planned as a means of determining the psychometric properties of the 9-item CDC HRQOL and its correspondence to the SF-36v2. All analyses used the full sample except when otherwise noted. Analyses were performed with the SAS 8.2 software package [11].

First, the underlying factor structure of the nine CDC HRQOL items was examined with a principal components analysis (PCA) with and without rotation. This involved performing the analysis on each sample (NC-FP-RN and MSK) and comparing the factor extractions and loadings. All items were included. After examining the factor results in each sample, the same PCA was used to examine the stability of the factor structure on the combined samples.

Next, a multi-trait/multi-method (MTMM) matrix was constructed from Pearson correlations to examine the extent and nature of shared variation in the identified CDC HRQOL scales and the PCS and MCS from the SF-36v2. Factors identified on the CDC HRQOL were used as scales. Scales were created by averaging the response values of the items that loaded on each factor. Items that loaded negatively were reverse scored, and those items that did not load strongly on any factor – or that loaded equally strongly on multiple factors, were not included in the scale scores. For this analysis, the general health item was not included in any of the CDC HRQOL scales because it has a different scaling metric and is identical to one of the SF-36v2 items. Cronbach's alphas were calculated to assess the internal consistency of these scales.

Finally, four multiple linear regression models were used to regress the CDC-HRQOL items on the PCS and MCS scales with and without the general health question in order to determine the relative contribution of the components of the CDC HRQOL items in predicting the SF-36v2. The eight CDC HRQOL items were simultaneously entered into the model as predictors of the PCS and MCS component of the SF-36v2. Results including the general health question in the model were compared with the results of excluding it in the model. The stability of responses to the general health question was evaluated overall and by the order in which the instruments were administered.

## Results

The participants' characteristics are presented in Table 1. First, the overall sample is described and then each sample's characteristics are presented separately, T-tests and chi-squared tests were computed to test for between-sample differences. Significant differences were found on several variables. Compared to the MSK sample, individuals in the NC-FP-RN tended to be younger, were more likely to be black, and less likely to be married.

**Table 1:** Sample Characteristics of adults with arthritis – North Carolina, 2002

Characteristic	Overall Sample (N = 1820)	NC-FP-RN Sample (N = 1139)	MSK Sample (N = 681)
Age^c ^Mean (SD)	56.9 (14.5)	54.5 (15.2)	60.7 (12.2)
Gender			
Female (N/%)	1383 (76%)	850 (75%)	533 (78%)
Male (N/%)	428 (24%)	280 (25%)	148 (22%)
Race^c^			
Black (N/%)	289 (16%)	234 (20%)	55 (8%)
Other (N/%)	102 (6%)	33 (3%)	69 (10%)
White (N/%)	1429 (78%)	872 (77%)	557 (82%)
Education ^c^			
Less than high school (N/%)	328 (18%)	242 (22%)	86 (13%)
High school graduate (N/%)	546 (31%)	337 (30%)	209 (32%)
More than high school (N/%)	907 (51%)	540 (48%)	367 (55%)
Marital Status^c^			
Married (N/%)	1160 (65%)	679 (60%)	481 (73%)
Not married (N/%)	633 (35%)	451 (40%)	182 (27%)
9-item CDC HRQOL Mean (SD) 4-item Healthy Days Core Module			
1. General health rating	3.3 (1.0)	3.3 (1.0)	3.4 (1.0)
2. Physical health not good (# days) ^c^	12.2 (11.1)	11.4 (11.0)	13.6 (11.0)
3. Mental health not good (# days)	8.7 (10.2)	8.9 (10.4)	8.3 (9.9)
4. Physical and/or mental health limited usual activities (# days)	8.1 (10.4)	7.8 (10.5)	8.7 (10.3)
5-item Healthy Days Symptoms Module			
5. Pain limited activities (# days) ^b^	12.2 (11.9)	11.4 (11.9)	13.6 (11.8)
6. Sad, blue or depressed (# days)	8.2 (10.1)	8.5 (10.3)	7.7 (9.7)
7. Worried, tense, or anxious (# days) ^a^	10.0 (10.9)	10.6 (11.1)	9.1 (10.4)
8. Not enough rest (# days)	13.4 (11.3)	13.5 (11.3)	13.2 (11.2)
9. Very healthy/full of energy (# days) ^b^	9.0 (10.4)	9.7 (10.5)	7.9 (10.0)
			
SF-36v2 Mean (SD)			
Physical component summary ^c^	37.4 (12.6)	39.1 (12.6)	34.6 (12.2)
Mental component summary ^a^	43.3 (9.6)	42.8 (9.7)	44.3 (9.5)

a = p < .01

b = p < .001

c = p < .0001 for North Carolina-Family Practice-Research Network (NC-FP-RN) and Musculoskeletal (MSK) sample differences based on t-test for age, SF-36v2 measures, CDC items, and X^2 ^for gender, race, and marital status

### Principal Components Analysis

PCA's were run separately on each sample (NC-FP-RN and MSK) to insure the stability of the factor structure. For the unrotated solution, the second of the first two eigenvalues was dramatically smaller for the NC-FP-RN sample (5.2 vs. 1.08) and for the MSK sample (5.12 vs. 1.30). In both samples, the items loaded at (an absolute value of) 0.65 or better on the first factor. With promax rotation, a two factor solution emerged in both samples. These results suggest that the factor structure remains the same across these patient samples. In addition, when the same analyses were run according to the order in which the SF-36v2 or CDC HRQOL measures were given, the results produced identical item-factor loadings. The eigenvalues were within 3/100^th ^of being the same value.

It was not surprising then that the results for the combined samples were very similar to those found in each sample. The unrotated PCA yielded two factors with eigenvalues greater than one. Although two factors were extracted, all of the items loaded at an absolute value of 0.66 or greater on the first factor (Table 2). The one-factor solution accounted for 57% of the total variance. The first eigenvalue was 5.16 and the second was 1.17. When a promax rotation was applied, a two-factor solution emerged. The variance explained by these factors was 25% and 21%, respectively. Four items capturing physical problems related to health (poor physical health, activities limited by physical and mental health, pain, and energy level) loaded on the first factor, as did the general health question. These loadings were all greater than an absolute value of 0.70. The three items addressing mental health-related symptoms (poor mental health, sad/depressed, and worried/tense) loaded more clearly (at 0.88 or higher) on the second factor. Only one item (not enough rest) loaded equally on both factors and therefore, was not considered a good measure of either construct. Table 2 presents the factor loadings for the unrotated and rotated solutions.

**Table 2:** Factor loadings for the CDC HRQOL for the overall sample of adults with arthritis

Item # and Item phrasing	Unrotated Factor Loadings		Rotated Factor Loadings (Promax)	
	Factor 1	Factor 2	Factor 1	Factor 2
5. pain limited activities (# days)^a^	0.77	-0.37	0.86	-0.01
2. physical health not good (# days)^a^	0.80	-0.34	0.84	0.04
1. General Health Rating	0.74	-0.35	0.82	0.00
9. very healthy/full of energy (# days)^a^	-0.66	0.29	-0.71	-0.02
4. physical and/or mental health limited usual activities (# days)^a^	0.78	-0.21	0.70	0.17
8. not enough rest (# days)^c^	0.69	0.06	0.37	0.41
6. sad, blue or depressed (# days)^b^	0.80	0.50	-0.01	0.94
3. mental health not good (# days)^b^	0.79	0.47	0.01	0.91
7. worried, tense, or anxious (# days)^b^	0.79	0.45	0.04	0.88

^a^Included on Factor 1, the CDC-HRQOL Physical Health Scale (CDC-PH)

^b^Included on Factor 2, the CDC-HRQOL Mental Health Scale (CDC-MH)

^c^Not included on either scale

### Multi-trait/Multi-method Matrix

Two scales were created from the PCA with rotation. The CDC HRQOL physical health scale (CDC-PH) included the four CDC items (minus the general health rating) that loaded on the first factor (Table 2). Although the general health item loaded on the first factor, it was not included because it would artificially inflate correlations with the SF-36v2, which contains the same item. The CDC HRQOL mental health scale (CDC-MH) included the three – CDC HRQOL items that loaded on the second factor (Table 2). All items included on these scales used the same 30-day metric. The one item that loaded negatively (very healthy/full of energy) was reverse-scored, and then the mean of the items in each scale was computed to represent the scale score. The Cronbach alpha reliability coefficient for B-PH was 0.84 and for CDC-MH was 0.91. When analyzed according to the order in which the instruments (SF-36v2 and CDC HRQOL were given), the results were nearly identical with the Cronbach alpha values falling within 1/100^th ^of the alphas for the overall sample.

Correlations were computed between each trait (physical health and mental health) and between each method of measurement (CDC HRQOL and SF-36v2). High scores on the SF-36v2 correspond to better functioning; whereas, high scores on CDC-PH and CDC-MH correspond to worse functioning. As a result, a strong negative correlation between the scales on the different instruments represents a strong positive relationship. In contrast, a strong negative relationship between scale scores on the same instrument would be represented by a positive correlation. The CDC-PH factor was more strongly correlated with the PCS (r = -.78, p < 0.0001) than the MCS (r = -.50, p < 0.0001), and the CDC-MH factor was correlated more strongly correlated with the MCS (r = -.71, p < 0.0001) than the PCS (r = -.35, p < 0.0001). The PCS and the MSC were weakly correlated (r = 0.23, p < 0.0001), but the CDC-PH factor was somewhat correlated with the CDC-MH factor (r = 0.58, p < 0.0001).

### Regression

All of the CDC HRQOL items were entered into the regression equation as predictors of the PCS and MCS scales and these results are presented in Table 3. With the general health rating included (same item as that in the SF-36v2 scales), the relative contribution of the CDC-HRQOL in predicting PCS was 73% (R^2 ^= .73) and when the item was removed was 65% (R^2 ^= .65). Whether the general health rating was included or removed, the relative contribution of the CDC HRQOL in predicting MCS was 56% (R^2 ^= .56).

**Table 3:** Regression of CDC HRQOL items (with and without the general health item) on the SF-36v2 Scales

CDC HRQOL item # and item phrasing	SF-36v2 PCS^a^		SF-36v2 MCS^b^	
	With general health item	Without general health item	With general health item	Without general health item
1. General health rating	-5.22	N/A	-0.17	N/A
2. Physical health not good (# days)	-0.17	-0.30	0.04	0.03
3. Mental health not good (# days)	0.12	0.13	-0.12	-0.12
4. Physical and/or mental health limited usual activities (# days)	-0.11	-0.17	-0.16	-0.16
5. Pain limited activities (# days)	-0.34	-0.39	0.09	0.08
6. Sad, blue or depressed (# days)	0.09	0.05	-0.33	-0.34
7. Worried, tense, or anxious (# days)	0.04	0.02	-0.15	-0.15
8. Not enough rest (# days)	0.02	0.02	-0.08	-0.08
9. Very healthy/full of energy (# days)	0.24	0.39	0.10	0.10

^a^Physical component summary

^b^Mental component summary

Responses to the General Health item appeared stable and were not significantly different depending on whether the CDC HRQOL or the SF-36v2 was administered first e.g., respondents who answered "excellent" on the first question tended to give the same response later in the survey. Correlations between the categorical responses on the SF-36v2 item and the CDC HRQOL item ranged from 0.88 to 0.95.

## Discussion

Arthritis has a tremendous impact on HRQOL. Thus, measuring HRQOL is an important part of assessing the burden of disease. The National Arthritis Action Plan (NAAP) in conjunction with the Arthritis Foundation supports using the CDC HRQOL modules to increase HRQOL surveillance in people with arthritis [12]. State and local efforts can then target populations who bear the greatest burden. In addition, the Healthy Days measures are explicitly incorporated into the goals for HRQOL in Healthy People 2010 [13]. This surveillance helps to target those high-risk individuals who would benefit from medical, self-help, and community-based interventions.

Mili et al. used the CDC HRQOL core module in the general population of 15 states and Puerto Rico and compared individuals with and without arthritis. For the 4-item Healthy Days Core module, participants with arthritis were three times more likely to report their general health as fair to poor and averaged more physical, mental, and overall unhealthy days than participants without arthritis [14]. Dominick et al. examined the CDC HRQOL modules using Medicare data on 41,467 older adults from Pennsylvania with and without arthritis. They reported the CDC's HRQOL modules were able to distinguish between those with and without arthritis as well as between the different types of arthritis (OA and RA) [15]. In another study by Currey et al., differences in HRQOL among diagnoses groups (OA, RA, and FM) that were established using condition-specific measures were mirrored on the 4-item Healthy Days Core Module in a clinical population with arthritis [16]. There appears to growing evidence of the advantages of using the CDC HRQOL modules in the clinic [17].

A CDC-funded validation study concluded that the CDC HRQOL modules are a reasonable alternative to the SF-36v2, but this study lacked the sample size to evaluate specific disease groups such as arthritis [10]. A more recent study by Abell et al. used the 2001 BRFSS data from all 50 states, the District of Columbia, and the US territories to assess the relationship between physical activity and HRQOL in people with arthritis. People with arthritis had more unhealthy days compared to those without arthritis. This study's main focus was however not on the psychometric properties of the CDC HRQOL items [18]. Therefore, the results of our study are an important contribution to the conceptualization of HRQOL in people with arthritis.

The underlying factor structure of the CDC HRQOL remained stable across the different patient samples. The samples differed from each other in two distinct ways, i.e., community-based vs. subspecialty and self-reported vs. physician-reported arthritis. The MSK participants are from musculoskeletal subspecialty clinics and they had a physician diagnosis of arthritis. The NC-FP-RN participants are from general practitioners across the state and had self-reported arthritis. Despite these distinctions the CDC HRQOL items remained stable when examined across these populations. Future studies on a population with physician-reported arthritis and physical and mental assessments that are not self-reported would provide further evidence for the validity of the 9-item CDC HRQOL because of potentially more accurate measures.

The unrotated solution suggests that when unconstrained, the mental and physical health-related problems of the CDC HRQOL are so correlated that they are not clearly distinguishable. In this case, a general health factor emerges. This general factor encompasses mental and physical health-related problems and this one-factor solution could be used as a general measure of HRQOL. When rotation is used, the two factors become distinguishable as correlated but distinct factors. A two-factor solution could be used to give more information about the contribution of physical problems and mental health problems to the overall score. These two factors will not be equally correlated for every individual or in every case, so to separate them gives more information.

In a MTMM analysis, the highest correlations are expected between the two different measures of the same trait, while the different trait/same method correlations are expected to be lower. These relationships were confirmed. Although the MTMM correlation was very low for CDC-MH with PCS (-0.35), the CDC-PH and MCS relationship was surprisingly strong (-0.50); this is counterintuitive because correlating different methods and different traits usually results in the lowest correlations. Another counterintuitive finding was the correlation between the two factors on the CDC HRQOL, which was relatively strong (0.58). These relationships suggest that there is a correlation between HRQOL related to physical and mental health problems, and the CDC HRQOL factors seem to reflect this more than the SF-36v2 factors.

Consistent with the factor analysis results of the CDC HRQOL, the general health item seems most closely related to scores on the physical health factor despite the strong correlation between physical and mental health problems. Whereas the inclusion of the general health rating increased the prediction of physical health, it made no difference in predicting mental health as measured on the SF-36v2. Although these results (regression) make the CDC HRQOL appear to be a good approximation of the SF-36v2, the physical and mental health factors are more highly correlated and therefore less distinct than the 'equivalent' SF-36v2 factors. In this study, the CDC PH and CDC MH are proxy measures of a proxy for HRQOL and therefore may include some drift away from validity.

One possible explanation for this drift away from validity is the use of the equal-weighting method employed to create the scale. Although, correlations between the weighted factor scores (from the PCA) and the unweighted means of the CDC PH and CDC MH were >0.99 (p < .0001) for both the overall sample and by sample thus supports the case that the weighted and the unweighted methods of scaling provide similar information. An alternative approach for future research might be to use differentially weighted items based on the relative strength of each item in relation to a standard measure of HRQOL. For example, the Healthy Days Core Module physically and mentally unhealthy days are widely used as single-item global measures of HRQOL. Compared to the global Core Module measures, the Optional Module measures are amendable causes of poor mental or physical HRQOL that allow monitoring of modifications in health programs [6]. Therefore, future studies might compare differentially weighted Optional Module measures with these global measures and/or with the summary index of unhealthy days.

In this study, there did not appear to be any respondent fatigue or ordering effects. Although, substantial differences would have been important (and, thus, our choice to alternate order the results based on alternating order didn't differ substantially from the results disregarding order. The factor structure for the subscales and the alphas for each subscale did not change according to test order.

The psychometric properties of the 9-item CDC HRQOL support its use in both community-based populations with self-reported arthritis and subspecialty-based populations with physician-reported arthritis. The factor structure of the CDC HRQOL remained stable across both these patient populations. The results of this study are helpful in instances where parsimony of items is important. The 4-item CDC-PH could be used when the primary goal of a project is to measure physical HRQOL; the 3-item CDC-MH could be used when the primary goal is to measure mental HRQOL. Both the CDC-PH and CDC-MH subscales demonstrated good internal consistency. The expected correlations in the MTMM supported the construct validity of the CDC-PH and CDC-MH subscales [19]. In summary, the CDC HRQOL appears to be a valid way to monitor the health-related quality of life of people with arthritis.

## Competing interests

The author(s) declare that they have no competing interests.

## Authors' contributions

SC provided conceptualization. RD and LC provided assistance with conceptualization. TM, EJ, and SC provided writing. RD and LC provided assistance with writing. TM, SC, and LC provided data collection. EJ provided analysis. TM, RD, and LC provided assistance with analysis. TM, EJ, SC, RD, and LC provided consultation (including review of manuscript before submission).
